# Direct Current Pulse Atmospheric Pressure Plasma Jet Treatment on Electrochemically Deposited NiFe/Carbon Paper and Its Potential Application in an Anion-Exchange Membrane Water Electrolyzer

**DOI:** 10.1021/acs.langmuir.4c01169

**Published:** 2024-07-01

**Authors:** Shuo-En Yu, Yu-Lun Su, I-Chih Ni, Yi-Cheng Chuang, Cheng-Che Hsu, Chih-I Wu, Yong-Song Chen, I-Chun Cheng, Jian-Zhang Chen

**Affiliations:** † Graduate School of Advanced Technology, National Taiwan University, Taipei City 106319, Taiwan; ‡ Institute of Applied Mechanics, National Taiwan University, Taipei City 106319, Taiwan; § Graduate Institute of Photonics and Optoelectronics and Department of Electrical Engineering, National Taiwan University, Taipei City 106319, Taiwan; ∥ Department of Mechanical Engineering and Advanced Institute of Manufacturing with High-Tech Innovations, National Chung Cheng University, Minhsiung, Chiayi 621301, Taiwan; ⊥ Department of Chemical Engineering, National Taiwan University, Taipei City 106319, Taiwan; # Advanced Research Center for Green Materials Science and Technology, National Taiwan University, Taipei City 106319, Taiwan

## Abstract

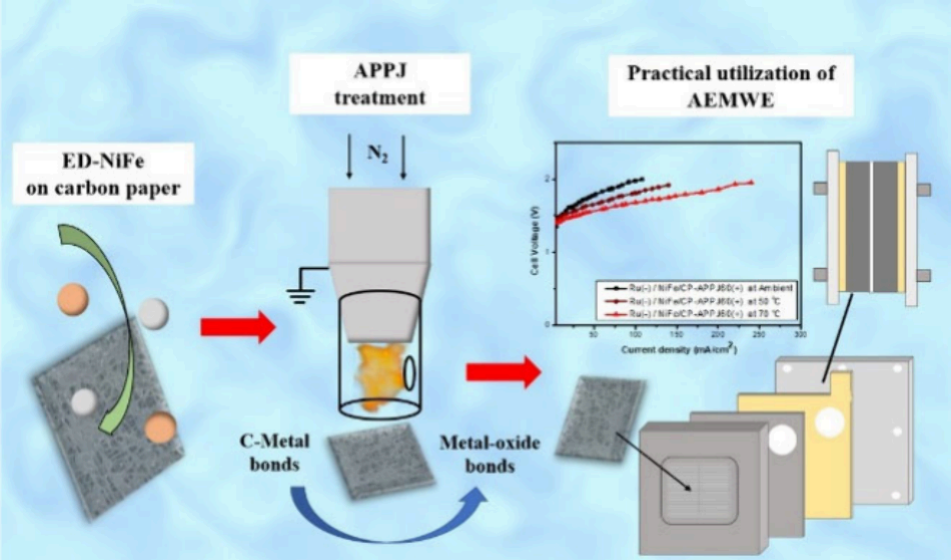

An atmospheric pressure plasma jet (APPJ) is used to process electrochemically deposited NiFe on carbon paper (NiFe/CP). The reactive oxygen and nitrogen species (RONs) of the APPJ modify the surface properties, chemical bonding types, and oxidation states of the material at the self-sustained temperature of the APPJ. The APPJ treatment further enhances the hydrophilicity and creates a higher disorder level in the carbon material. Moreover, the metal carbide bonds of NiFe/CP formed in the electrochemical deposition (ED) process are converted to metal oxide bonds after APPJ processing. The potential application of APPJ treatment on NiFe/CP in alkaline water electrolysis is demonstrated. With more oxygen-containing species and better hydrophilicity after APPJ treatment, APPJ-treated NiFe/CP is applied as the electrocatalyst for the oxygen evolution reaction (OER) in alkaline water electrolysis. APPJ-treated NiFe/CP is also used in a custom-made anion-exchange membrane water electrolyzer (AEMWE); this should contribute toward realizing the practical large-scale application of AEM for hydrogen production.

## Introduction

Atmospheric pressure plasma (APP) functions at atmospheric pressure and, thus, does not require a vacuum chamber or a complex pumping system. Consequently, APP technology involves lower maintenance costs and a straightforward equipment setup, and thus, it has attracted much research attention.^1,2^ APP is an economical and ecofriendly processing method, and it can be generated by various mechanisms. Common methods for producing APP, include an atmospheric pressure plasma jet (APPJ), corona discharge, dielectric barrier discharge (DBD), and transferred arc.^1,3^ These techniques can be used to easily generate APP with highly reactive oxygen and nitrogen species (RONs)^4^ under atmospheric pressure conditions, and they have been widely utilized in various application fields, such as biomedicine,^5,6^ surface modification,^7,8^ material etching,^9^ and nanomaterial fabrication.^10^ Furthermore, APPs have also been applied in the field of energy devices^8^ for the fabrication of supercapacitors,^11^ batteries,^12^ solar cells,^13^ and water electrolysis electrocatalysts.^14^

APPJ treatment has been applied as a rapid process to synthesize nanomaterials and oxides while modifying the surface properties.^15^ An APP is reactive in carbon-based materials,^16^ such as graphene,^11^ carbon nanotubes,^17^ and carbon fibers.^18^ Carbon-based materials having low cost, great thermal and chemical stability, and electrical conductivity^19^ are commonly used in energy devices. Earth-abundant transition metals, like Ni, Fe, and their oxide species, that have great electrochemical properties are also extensively used in energy storage devices^20−22^ and electrocatalysts.^23−25^

Hydrogen power is considered a form of clean energy and has thus attracted considerable research interest in recent years. Among various methods for producing hydrogen, water electrolysis shows great potential. In water electrolysis, the hydrogen evolution reaction (HER) and oxygen evolution reaction (OER) occur at the cathode and anode, respectively, with almost zero carbon emissions during hydrogen production with green power sources and simple equipment.^26,27^ The OER is considered more sluggish than the HER owing to the larger number of reaction pathways and more complex electrochemical kinetics.^28^ Consequently, in water electrolysis, an electrocatalyst that improves the OER is considered as one of the important strategies for increasing the electrolysis performance.^23,29^ However, expensive materials, such as IrO_2_, remain dominant in OER catalysts.^30^ High material costs have thus limited the large-scale application of water electrolysis.^31^ Therefore, low-cost and Earth-abundant materials need to be developed for use as electrocatalysts for the OER.^32^ NiFe-based materials, including alloys, oxides, and composites, have been studied extensively as OER catalysts because of their advantages, such as catalyst ability, low cost, and stability.^21,23,24^ Recent studies have highlighted the anion-exchange membrane water electrolyzer (AEMWE) as one of the hydrogen production devices.^33−35^ AEMWE affords the advantages of traditional alkaline water electrolysis and proton-exchange membrane water electrolysis (PEMWE), and it operates in a relatively non-corrosive alkaline environment at a lower working temperature and allows the use of a non-precious metal as the electrocatalyst.^33,35,36^ However, AEMWEs have thus far been applied only in laboratories. Further studies of AEMWEs are needed to facilitate their large-scale practical application.

In this study, we use an electrochemical deposition (ED) process to deposit NiFe on carbon paper (CP) with APPJ post-treatment. After APPJ treatment, the bonding status of metal carbides is transformed and the surface properties were altered. This study discusses the effect of APPJ treatment and demonstrates the potential application of APPJ-processed NiFe/CP as an electrocatalyst in AEMWE. A custom-made AEMWE is tested with the APPJ-treated NiFe/CP electrocatalyst. APPJ-treated NiFe/CP is used as the gas diffusion layer (GDL) of the anode, which catalyzes the OER. Further, Ru, which is considered one of the alternatives to the Pt catalyst for the HER, is grown on CP by the traditional hydrothermal method as the GDL of the cathode.

## Experimental Section

### Pretreatment of the CP

A CP substrate having a size of 5 × 5 cm and thickness of 0.43 mm (CeTech) was cleaned using a plasma cleaner. The low-pressure plasma pretreatment with 5% O_2_ and 95% Ar was aimed at removing contamination while improving the hydrophilicity of the CP. The low-pressure plasma pretreatment was performed at a power of 11 W with a processing time of 60 s.

### Preparation of NiFe/CP

The plasma-treated CP was immersed in an electroplating solution produced by dissolving 1 g of Ni(NO_3_)_2_·6H_2_O and 1.4 g of Fe(NO_3_)_3_·9H_2_O in 150 mL of ethanol.^37^ CP is used as both the anode and the cathode in the ED system, and the distance between the two electrodes is 2.5 cm. The NiFe ED process is performed by applying 10 V for 600 s. ED NiFe/CP was first dried under ambient conditions for 1 h and then further dried in an oven at 60 °C for 1 h.

### APPJ Treatment on NiFe/CP

**Figure 1:**
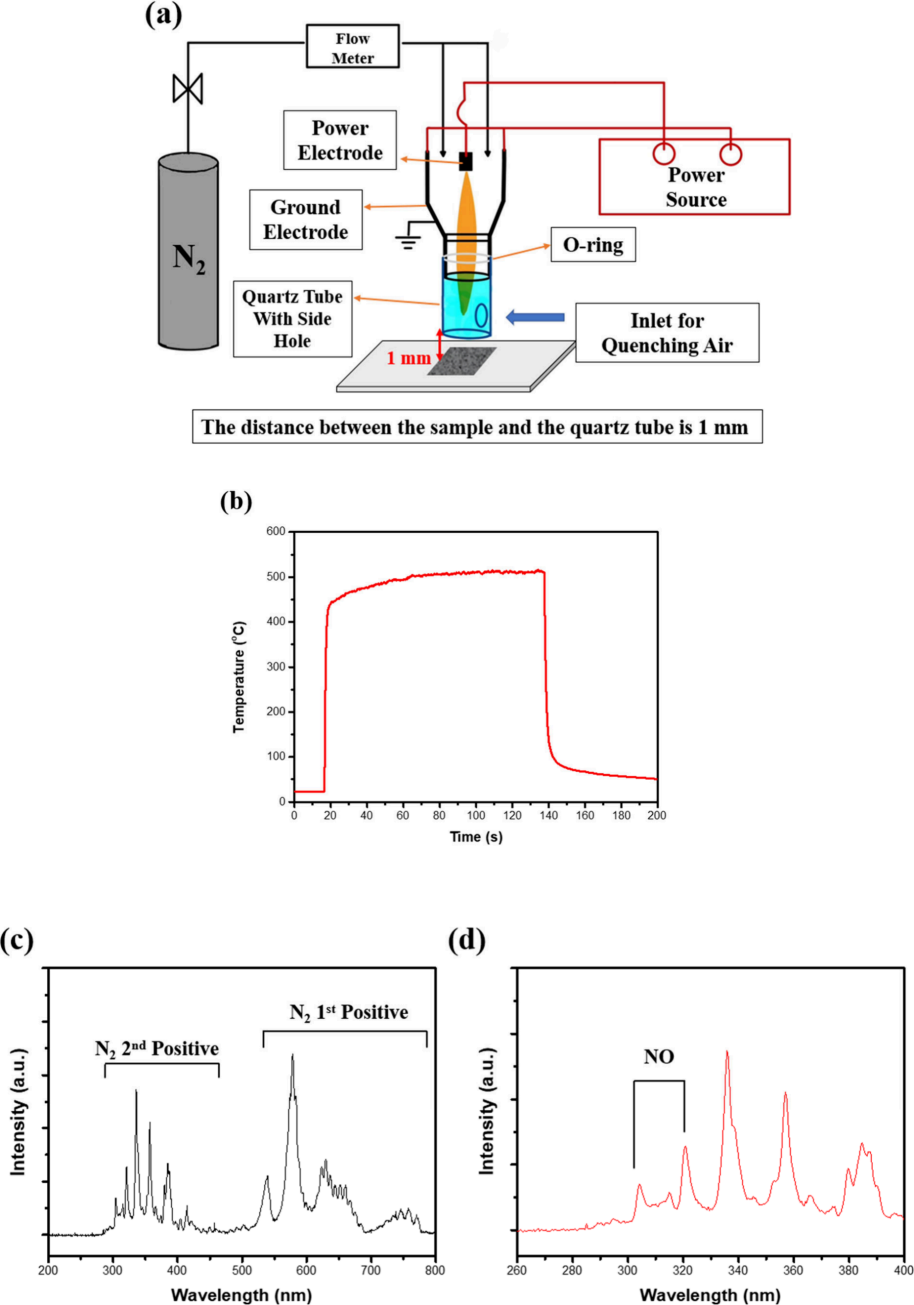
(a) Schematic illustration of the APPJ setup, (b) temperature evolution in the APPJ process, and (c and d) OES of the APPJ.

A direct current (DC) pulse nitrogen APPJ was used to post-treat NiFe/CP. Figure 1a shows the setup of the APPJ. The APPJ operation parameters are as follows: gas flow rate of nitrogen, 46 standard liters per minute (slm); DC power supply (before transformer) voltage, 275 V; and on/off duty cycle, 7/33 μs. At the downstream position of APPJ, a quartz tube (4.8 cm in length and 3 cm in inner diameter) with a side hole was installed to control the quenching air flow from the ambient and to introduce more oxygen from ambient air by the Bernoulli effect.^38^ This could generate more RONs. Figure 1b shows the evolution of the substrate temperature as measured by a K-type thermocouple (OMEGA Engineering, Norwalk, CT, U.S.A.). The temperature can reach around 500 °C in 60 s. The sample was heated directly by the plasma jet, and no extra heating device was used in this experiment. After turning on the APPJ, the plasma jet reached the sample and reactive plasma species reacted with the sample. Also, the sample was heated by the plasma jet, and the sample temperature is therefore rapidly increased to a plateau. After the APPJ is turned off, the heating stopped and the temperature rapidly decreased. The estimated power output of this APPJ was ∼700 W.^39^ More characterization of plasma generated by this APPJ equipment has been reported in the literature.^40,41^ The NiFe/CP sample was treated by APPJ for 60 s with a distance from the quartz tube of 1 mm (the sample is denoted as NiFe/CP-APPJ60). Optical emission spectroscopy (OES) of the APPJ was obtained using a spectrometer (SP2500i, Princeton Instruments), as depicted in panels c and d of Figure 1. In OES of the APPJ, intense signals corresponding to N_2_ first positive B^3^∏_g_ → A^3^∑_u_^+^ and N_2_ second positive C^3^∏_u_ → B^3^∏_g_ indicate the presence of energetically excited N_2_ molecules.^41,42^ Furthermore, in the shorter wavelength region, signals attributed to NO molecules can be observed, suggesting that oxygen from the ambient air introduced by the side hole reacts with the nitrogen reactive species in the APPJ, resulting in NO molecules.^41,42^

### Material Characterization

A goniometer was used to analyze the water contact angles (model 100SB, Sindatek). Field-emission scanning electron microscopy (SEM, JEOL 6500F Oxford) with energy-dispersive X-ray spectroscopy (EDS) was used to analyze the morphology and element contents. X-ray diffraction (XRD, Bruker D8 Discover) was performed with Cu Kα radiation. X-ray photoelectron spectroscopy (XPS, Sigma Probe, Thermo VG Scientific, Waltham, MA, U.S.A.) with an Al Kα source (1486.6 eV) was conducted to study the element contents, surface chemical bonding, and oxidation state. A vibrational spectroscopic imaging system (Nanonics/MultiVeiw 4000) was used to perform confocal Raman microscopy (CRM) analysis (laser source of 532 nm).

### Electrochemical Measurements

Electrochemical measurements were performed using an electrochemical workstation (Autolab PGSTAT204, Metrohm, Utrecht, Netherlands) under ambient conditions. A conventional three-electrode setup was used in a 1 M KOH electrolyte, with Ag/AgCl as the reference electrode, Pt as the counter electrode, and a working electrode composed of CP. The potential was transformed into the relative potential corresponding to the reversible hydrogen electrode (*E*_RHE_) using the Nernst equation^43,44^
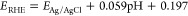
where *E*_RHE_ represents the potential of the reversible hydrogen electrode and *E*_Ag/AgCl_ denotes the measured potential against the Ag/AgCl electrode.^32^ Linear sweep voltammetry (LSV) was conducted at a scan rate of 5 mV/s. Electrochemical impedance spectroscopy (EIS) measurements were carried out over a frequency range from 10 kHz to 0.1 Hz. Cyclic voltammetry (CV) was performed with a potential scan speed of 20–300 mV, spanning a range from 0.25 to 0.05 V relative to the Ag/AgCl electrode.

### AEMWE

**Figure 2:**
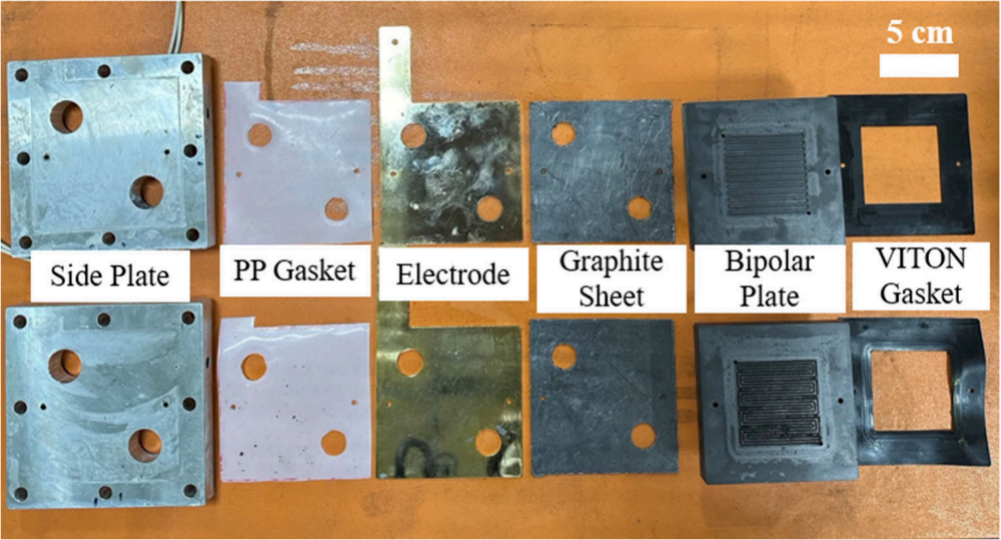
Components of custom-made AEMWE.

The composition of the custom-made AEMWE with a symmetric structure is shown in Figure 2. The electrolyzer comprises aluminum alloy side plates with heaters, polypropylene gaskets, gold-coated copper electrodes, flexible graphite sheets, graphite bipolar plates, and VITON gaskets. The aluminum alloy side plates are used to fix the entire AEMWE cell and facilitate heat conduction. The electrolyte flow and water electrolysis reactions occur in the graphite bipolar plates and gas diffusion layer. The GDLs with an electrocatalyst in 5 × 5 cm were fixed in the VITON gaskets. The 6 × 6 cm anion-exchange membrane (Fumasep FAA-3-PK-130, soaked in 1 M KOH for 24 h) covered the GDLs and separated the two sides of the anode and cathode. The electrolyte (1 M KOH) was supplied to both the anode and cathode of the system through a peristaltic pump operating at a flow rate of 10 mL/min.

The GDL of the anode was prepared according to a flowing process. The as-deposited NiFe/CP was treated using APPJ for 60 s. A traditional hydrothermal method was applied to deposit ruthenium (Ru),^45−47^ considered one of the alternative catalysts to Pt, on the cathode GDL. The pretreated carbon fiber paper was immersed in a solution with 5 mmol of RuCl_3_·3H_2_O and 80 mL of ethylene glycol dissolved in 80 mL of deionized water in a Teflon autoclave. The Teflon autoclave was heated at 160 °C for 16 h to obtain the GDL of the cathode.

## Results and Discussion

### Water Contact Angle

**Figure 3:**
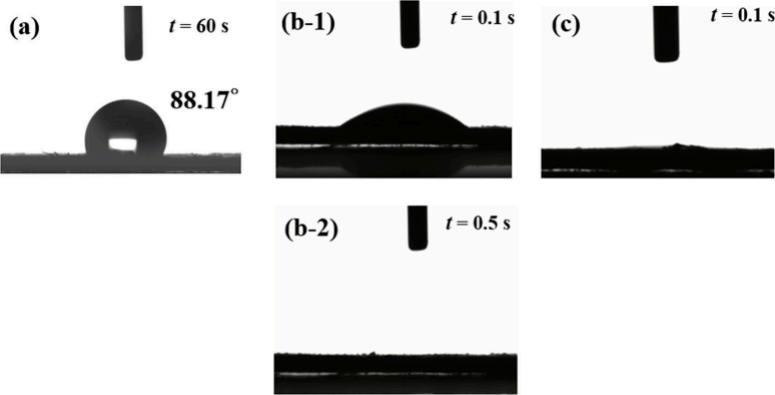
Water contact angles of (a) pretreated CP, (b-1) NiFe/CP, and (c) NiFe/CP-APPJ60 right after the droplet was dispensed and (b-2) test of droplets penetrating NiFe/CP.

For the surface properties of the material, the water contact angle was used as a preliminary analysis, as shown in Figure 3. Pretreated CP was relatively hydrophobic, with a water contact angle of 88.17°. As-deposited NiFe/CP was hydrophilic, and the testing droplet penetrated NiFe/CP in 0.5 s. For NiFe/CP-APPJ60, the testing droplet penetrated the substrate immediately, indicating improved hydrophilicity. APPJ treatment introduced more oxygen-containing species^4,48^ on the surface, further improving the hydrophilicity. Great hydrophilicity is beneficial to the water electrolysis. The better hydrophilicity could offer a better contact interface for the electrode and electrolyte as well as increase the detachment rate of the gas bubble during the water electrolysis process.^32,49^

### SEM with EDS Mapping

**Figure 4:**
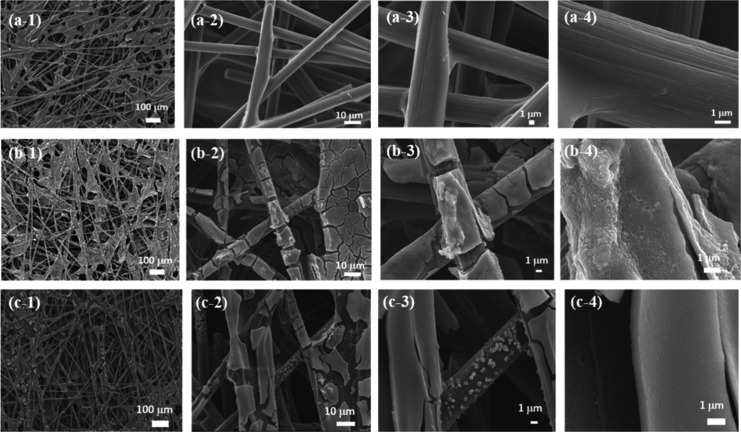
SEM images of CP with (a-1) 100×, (a-2) 1000×, (a-3) 3000×, and (a-4) 10000× magnification, NiFe/CP with (b-1) 100×, (b-2) 1000×, (b-3) 3000×, and (b-4) 10000× magnification, and NiFe/CP-APPJ60 with (c-1) 100×, (c-2) 1000×, (c-3) 3000×, and (c-4) 10000× magnification.

Figure 4 shows the SEM images. The CP shows a porous structure with carbon fibers in Figure 4a. Figure 4b shows the NiFe sheets and particles formed on the carbon fibers after the ED process. According to the SEM image of NiFe/CP-APPJ60 in Figure 4c, the NiFe material was still well-attached to the carbon fiber paper, indicating that the APPJ treatment did not cause severe damage to NiFe/CP. Moreover, some parts of the sample treated by APPJ exhibit a smoother and more separated morphology under macroscopic observation. We suspect that the samples treated with APPJ might undergo some peeling of small sheets, which are unstable and clustered on the carbon paper fibers during the electrochemical deposition process, influenced by the high-energy particles and reactive species in the plasma. However, this does not affect the application and performance of NiFe/CP-APPJ60 as the GDL.

**Figure 5:**
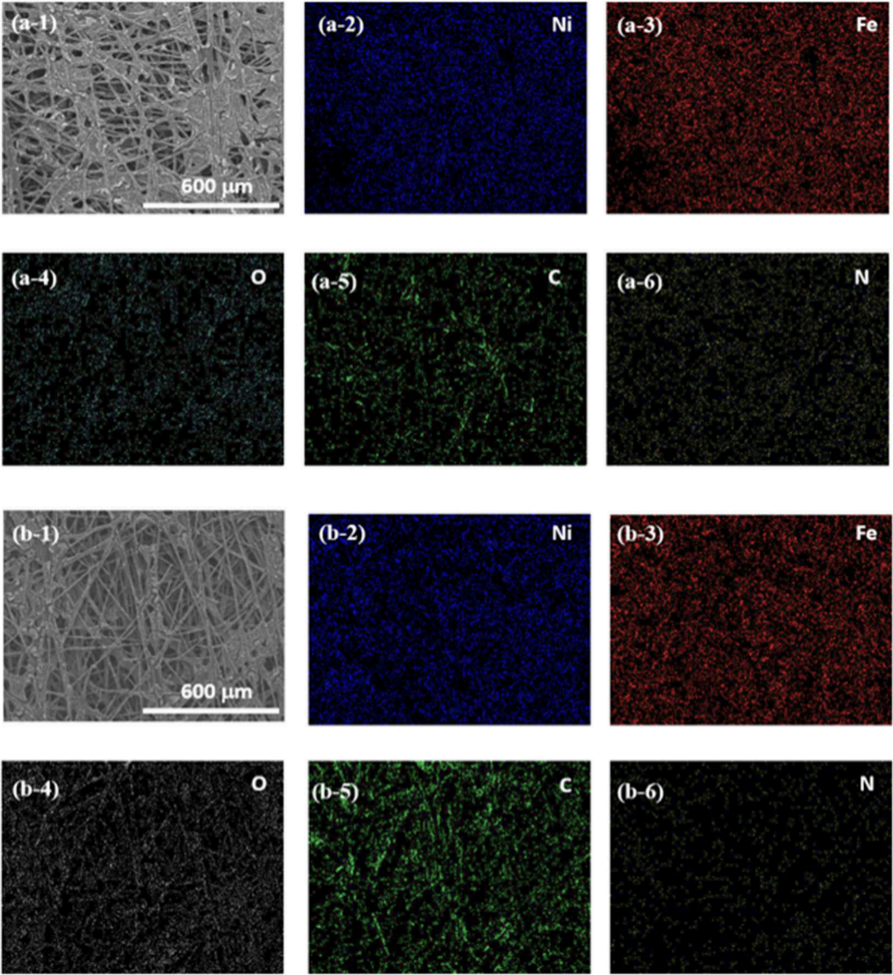
EDS mapping results of (a) NiFe/CP and (b) NiFe/CP-APPJ60. Panels a-2 and b-2, a-3 and b-3, a-4 and b-4, a-5 and b-5, and a-6 and b-6 are the mapping results of Ni, Fe, O, C, and N, respectively.

To further investigate the effect of APPJ treatment on NiFe/CP, EDS mapping was performed. Figure 5a shows the mapping result of as-deposited NiFe/CP, and Figure 5b presents the analysis results of NiFe/CP-APPJ60. Both NiFe/CP and NiFe/CP-APPJ60 exhibited signals of elements C, N, O, Ni, and Fe. However, the results showed that the nitrogen content decreased after APPJ treatment, as discussed below.

### XPS Analysis

**Figure 6:**
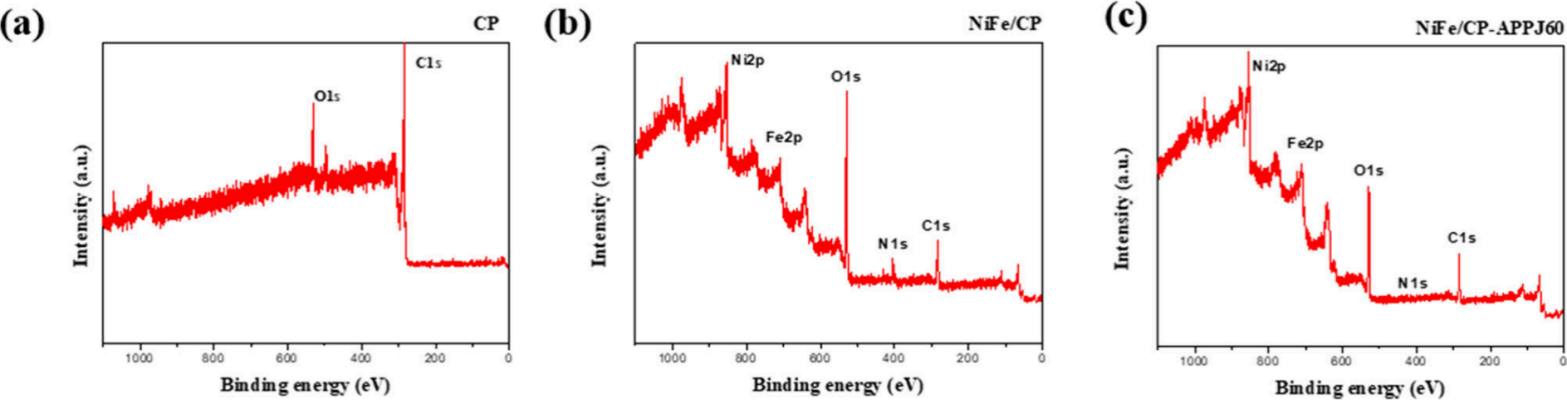
Full survey scan XPS spectra of (a) CP, (b) NiFe/CP, and (c) NiFe/CP-APPJ60.

XPS is performed to analyze the element contents, oxidation states, and chemical bonding types. Figure 6 shows the survey-scanned XPS spectra of CP, NiFe/CP, and NiFe/CP-APPJ60. The signals of Ni and Fe in the full survey spectra indicated that NiFe was deposited on the CP, and the signal of N decreased after APPJ treatment.

**Figure 7:**
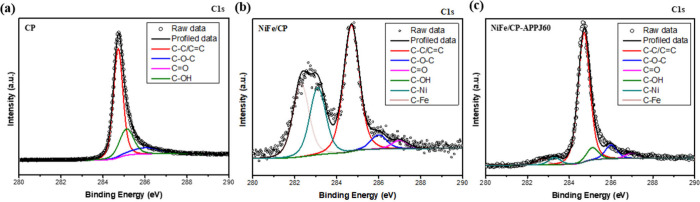
Fine-scan XPS spectra of C 1s for (a) CP, (b) NiFe/CP, and (c) NiFe/CP-APPJ60.

Figure 7 presents the bonding type in the C 1s XPS spectra. The XPS spectra of three samples, CP, NiFe/CP, and NiFe/CP-APPJ60, have a main peak at 284.7 eV for C–C/C=C bonds.^50,51^ Notably, untreated NiFe/CP presents a strong signal of the peak for carbide at 281–283 eV,^51−53^ including the C–Ni and C–Fe bonding. After the APPJ treatment, the signal of metal carbide decreased significantly, indicating that the bonding types of Ni and Fe were changed by the APPJ treatment. Table 1 shows the percentage of each different bonding type based on the XPS data. The percentages of C–Ni and C–Fe bonds in as-deposited NiFe/CP are 23.34 and 24.73%, respectively. However, the percentage of both C–Ni and C–Fe decreased to around 3–4% after the APPJ treatment.

**Table 1:** Percentage of Different Bonding Types of C 1s for Each Sample

C 1s	CP (%)	NiFe/CP (%)	NiFe/CP-APPJ60 (%)
C–C/C=C	64.81	44	74.38
C–O–C	11.56	4.95	7.96
C=O	0.12	2.88	3.3
C–OH	23.51		6.99
C–Ni		23.24	4.07
C–Fe		24.73	3.29

**Figure 8:**
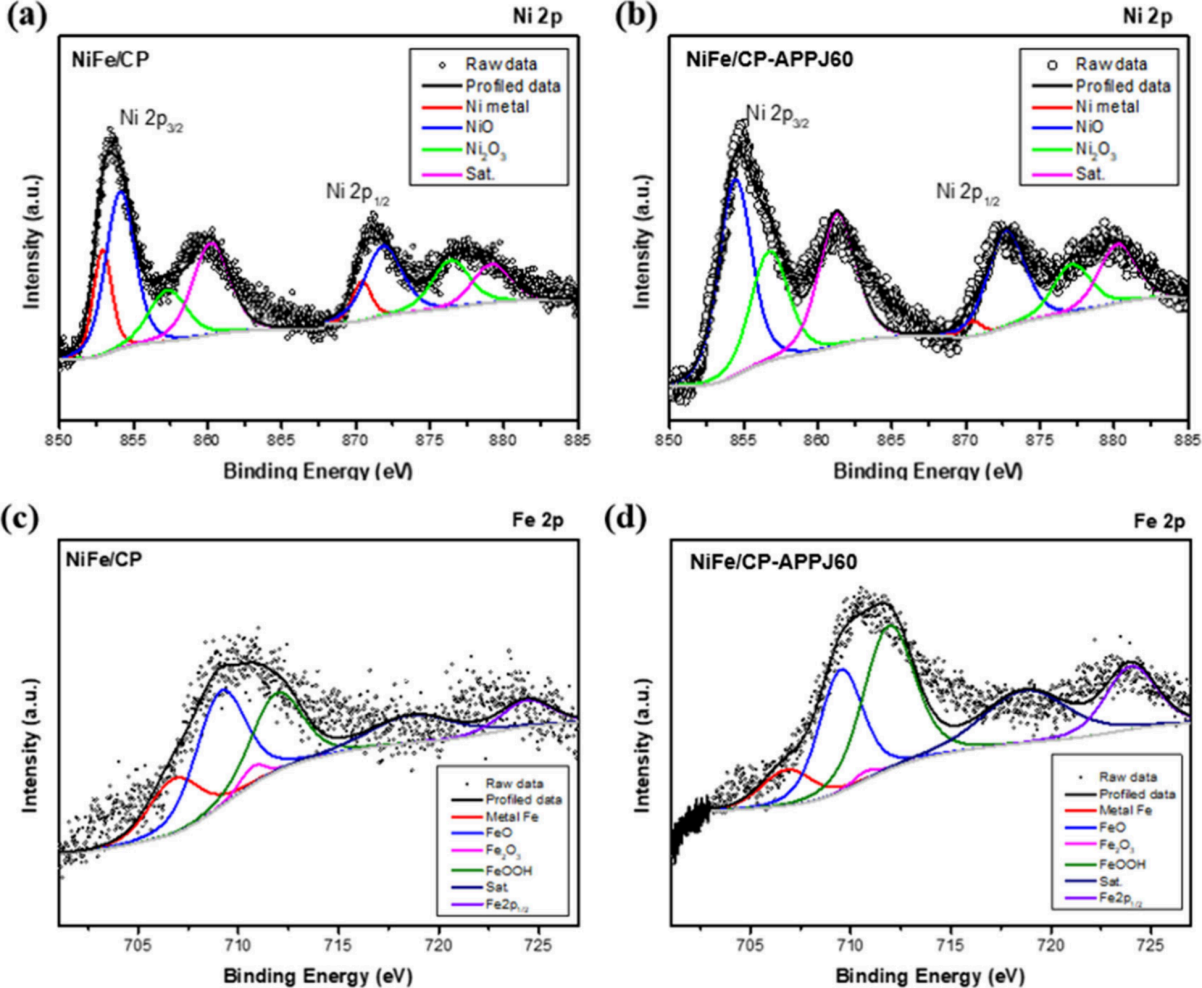
Fine-scan XPS spectra of Ni 2p for (a) NiFe/CP and (b) NiFe/CP-APPJ60. Fine-scan XPS spectra of Fe 2p for (c) NiFe/CP and (d) NiFe/CP-APPJ60.

To confirm the chemical bonding and oxidation states of Ni and Fe, the high-resolution spectra of Ni 2p and Fe 2p were analyzed. The profiled data of the Ni 2p orbital of NiFe/CP and NiFe/CP-APPJ60 are shown in panels a and b of Figure 8, respectively. In comparison to the Ni 2p curves of NiFe/CP and NiFe/CP-APPJ60, the curve of NiFe/CP-APPJ60 presents a slight rightward shift. A closer examination of the bonding type and oxidation state revealed that the amount of nickel oxide species, such as NiO and Ni_2_O_3_, increased after the APPJ treatment. The peaks are located at around 852.6, 854.5, and 856.1 eV in Ni 2p_3/2_, corresponding to metallic Ni, NiO, and Ni_2_O_3_, respectively.^32,51,54^ These results suggest that the oxidation states of Ni^2+^ and Ni^3+^ increased and that of metallic Ni^0^ decreased after the APPJ treatment. Further, the Fe 2p orbital was deconvoluted for investigating the chemical bonding. Panels c and d of Figure 8 present the chemical state of the Fe element. The APPJ treatment increased oxide species, including FeO, Fe_2_O_3_, and FeOOH, with the corresponding peak located at 709.6, 710.7, and 711.9 eV, respectively.^51,55,56^ Moreover, the contents of metallic Fe and Fe^0^, corresponding to the peak at 706.7 eV, also decreased. Table 2 presents the changes in the bonding type and oxidation state of Ni and Fe metals. After the APPJ treatment, the content of metallic Ni (Ni^0^) significantly decreased from 20.03 to 1.81% and the content of metallic Fe (Fe^0^) decreased from 21.52 to 12.03%. On the basis of the XPS analysis of Ni 2p and Fe 2p, the APPJ treatment introduced more oxygen-containing species to NiFe/CP. As a result, the oxidation of Ni caused by APPJ seemed more significant than that of Fe.

**Table 2:** Percentage of Different Bonding Types of Ni 2p and Fe 2p for Each Sample

Ni 2p	NiFe/CP (%)	NiFe/CP-APPJ60 (%)
Ni metal	20.03	1.81
NiO	52.80	61.92
Ni_2_O_3_	27.17	36.27

Fe 2p	NiFe/CP (%)	NiFe/CP-APPJ60 (%)
Fe metal	21.52	12.30
FeO	44.30	35.00
Fe_2_O_3_	3.53	1.89
FeOOH	30.65	50.81

**Figure 9:**
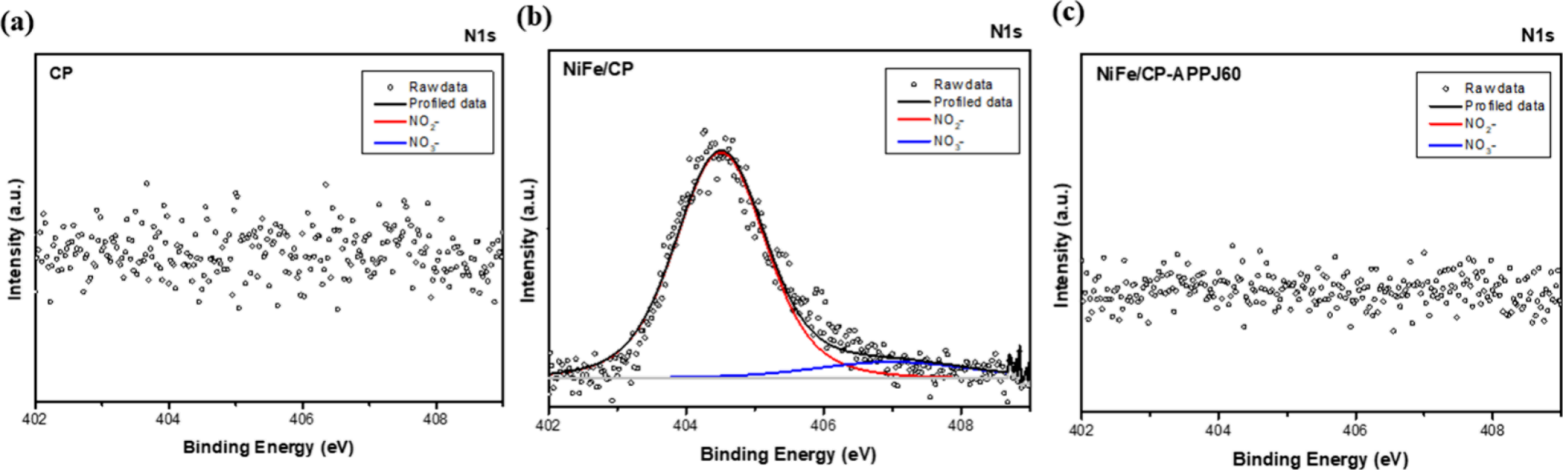
Fine-scan XPS spectra of N 1s for (a) CP, (b) NiFe/CP, and (c) NiFe/CP-APPJ60.

As discussed in the EDS analysis, the nitrogen content decreased after APPJ treatment. The high-resolution XPS spectrum of N 1s (Figure 9) shows the chemical state of nitrogen. In the sample of CP and NiFe/CP-APPJ60, the signal of N 1s was very weak. However, as-deposited NiFe/CP showed the signal of the N 1s peak. The peaks at around 404.2 and 407 eV correspond to NO_2_^–^ and NO_3_^–^, respectively. NO_2_^–^ and NO_3_^–^ may be produced in the solution used for the electrochemical deposition process performed in mixed nickel nitrate and iron nitrate solution. Then, they were removed by the APPJ treatment, resulting in the weak signal of the N atom in the EDS and XPS results.

### Raman Spectrum

**Figure 10:**
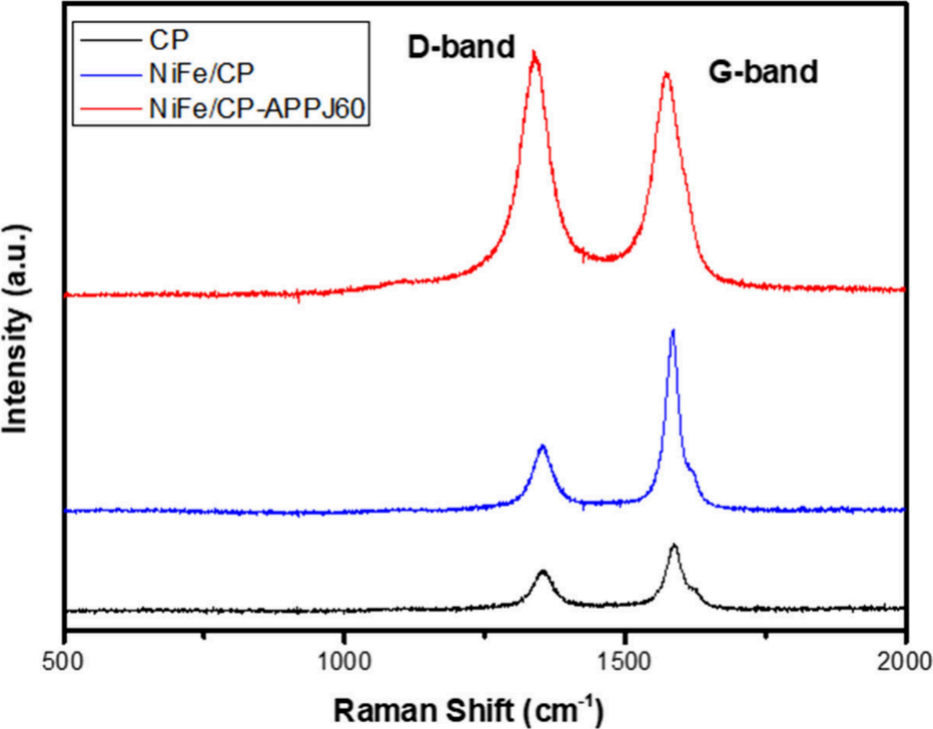
Raman spectra of CP, NiFe/CP, and NiFe/CP-APPJ60.

Raman spectroscopy was used to check the effect of APPJ treatment on the CP substrate. The G and D bands of the carbon material located at 1350 and 1580 cm^–1^ (Figure 10) represent the characteristics of the disorder and graphitic phase in the carbon material, respectively.^53,57^ In the Raman spectra, APPJ-processed NiFe/CP-APPJ60 shows a significantly higher D-band value than those of CP and NiFe/CP. The *I*_D_/*I*_G_ ratio could be used to quantify the defect level in the carbon material.^53,57,58^ The *I*_D_ and *I*_G_ values are 0.95, 0.84, and 1.024 in CP, NiFe/CP, and NiFe/CP-APPJ60, respectively. The APPJ treatment creates a higher level of disorder in NiFe/CP-APPJ60, possibly owing to the broken metal–carbon bonding resulted from the high-energy plasma species in the APPJ.^32,45^ The above-mentioned material reactions could be caused by heat, plasma reactive species bombardment of the plasma jet, or the synergetic effect of both. The real mechanisms require more theoretical and experimental investigations.

### Electrochemical Measurement

**Figure 11:**
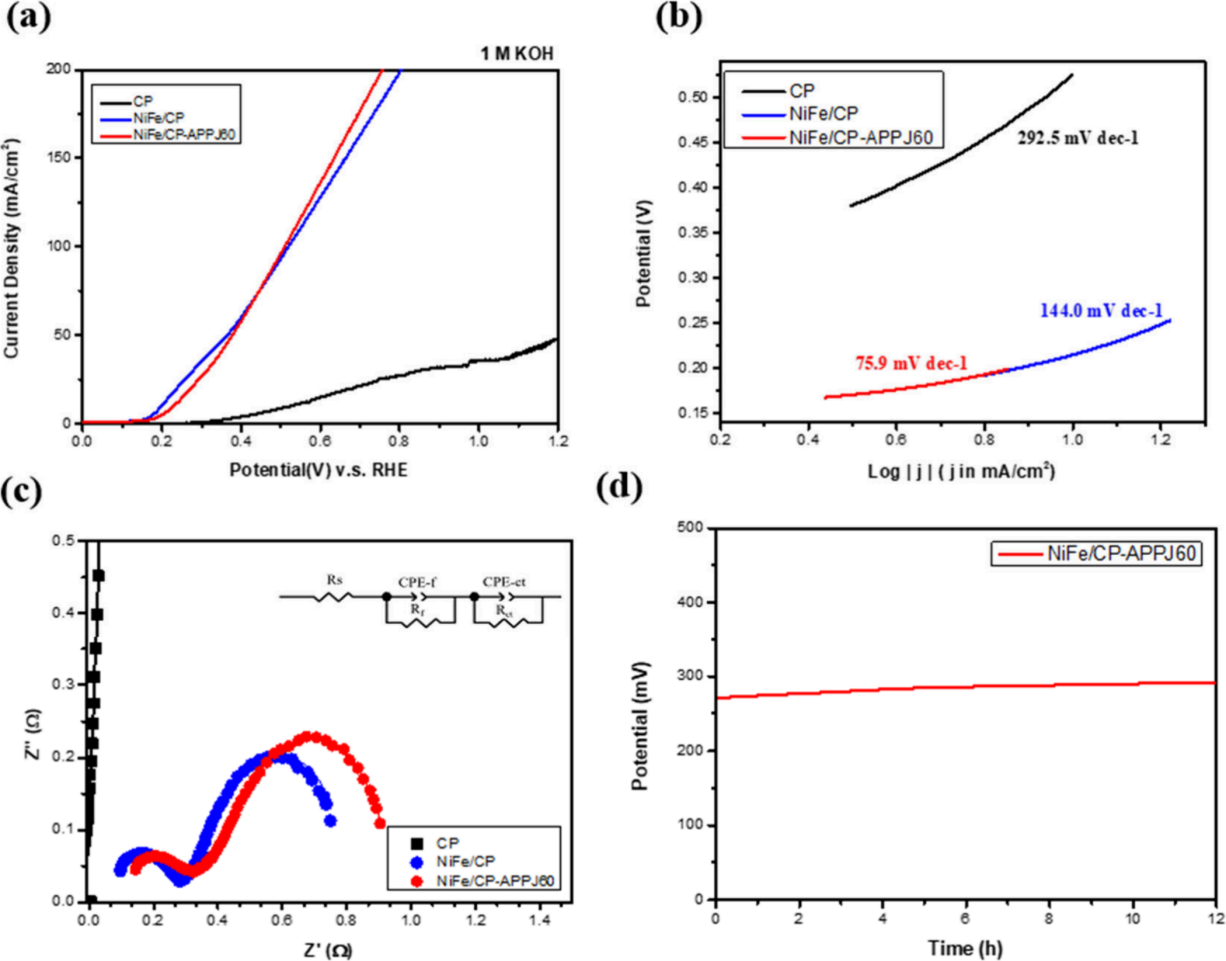
(a) LSV OER polarization curves in 1 M KOH, (b) Tafel slope plots, (c) Nyquist plots at an overpotential of 400 mV versus RHE, and (d) stability test of NiFe/CP-APPJ60.

Electrochemical measurements are performed in an alkaline environment of 1 M KOH to characterize the electrochemical properties and kinetics of the OER in alkaline water electrolysis. Figure 11 shows electrochemical characterization. Because of the complicated reaction pathways of the OER, an electrocatalyst with high activity in the OER could reduce the voltage required for electrolysis. In this regard, the overpotential (η), Tafel slope, and charge transfer resistance (*R*_ct_) are important parameters to determine the catalytic ability of the materials. The measurement of the overpotential at a higher current density can be closer to the practical usage situation of the water electrolysis system. Therefore, the LSV measurement was performed to calculate the overpotential of NiFe/CP and NiFe/CP-APPJ60. On the basis of the LSV results, both NiFe/CP and NiFe/CP-APPJ60 showed a significantly decreased overpotential in comparison to that of CP. NiFe/CP-APPJ60 (510.5 mV at 100 mA/cm^2^) presented a lower overpotential than that of NiFe/CP (520.2 mV at 100 mA/cm^2^), as shown in Table 3. Moreover, a lower Tafel slope signifies that a smaller voltage is required for an increase in the current, leading to decreased energy consumption.^59,60^ The Tafel slope of NiFe/CP-APPJ60 (75.9 mV dec^–1^) was lower than that of NiFe/CP, demonstrating its better catalytic performance. EIS analysis was performed at a potential of 400 mV versus the reversible hydrogen electrode (RHE) to investigate the OER kinetics of the interface between the electrode and electrolyte. The OER Nyquist plot and equivalent circuit for data fitting are shown in Figure 11c. The equivalent circuit includes the ohmic electrolyte resistance (*R*_s_), film porous structure resistance (*R*_f_), and *R*_ct_.^29,61^ The calculated values of *R*_f_ and *R*_ct_ are shown in Table 4. NiFe/CP-APPJ60 has a *R*_f_ of 0.26 Ω and a *R*_ct_ of 0.86 Ω, and NiFe/CP has a *R*_f_ of 0.47 Ω and a *R*_ct_ of 0.83 Ω. Overall, both NiFe/CP and NiFe/CP-APPJ60 showed lower resistance than that of CP. Furthermore, the stability of NiFe/CP-APPJ60 was tested for 12 h under a current density of 10 mA/cm^2^, as shown in Figure 11d. The overpotential was changed from 0.27 to 0.29 V with an increase of approximately +7.4% after the 12 h continuous measurement.

**Table 3:** Overpotential at Different Current Densities (mV)

electrocatalyst	at 10 mA/cm^2^	at 50 mA/cm^2^	at 100 mA/cm^2^	at 150 mA/cm^2^	at 200 mA/cm^2^
CP	613.9				
NiFe/CP	200.5	366.2	520.2	664.2	805.5
NiFe/CP-APPJ60	230.12	378.7	510.5	634.6	757.6

**Table 4:** EIS Analysis and 2*C*_dl_ Value of NiFe/CP and NiFe/CP-APPJ60

sample	*R*_f_ (Ω)	*R*_ct_ (Ω)	2*C*_dl_ (mF/cm^2^)
NiFe/CP	0.47	0.83	1.79
NiFe/CP-APPJ60	0.26	0.86	2.05

**Figure 12:**
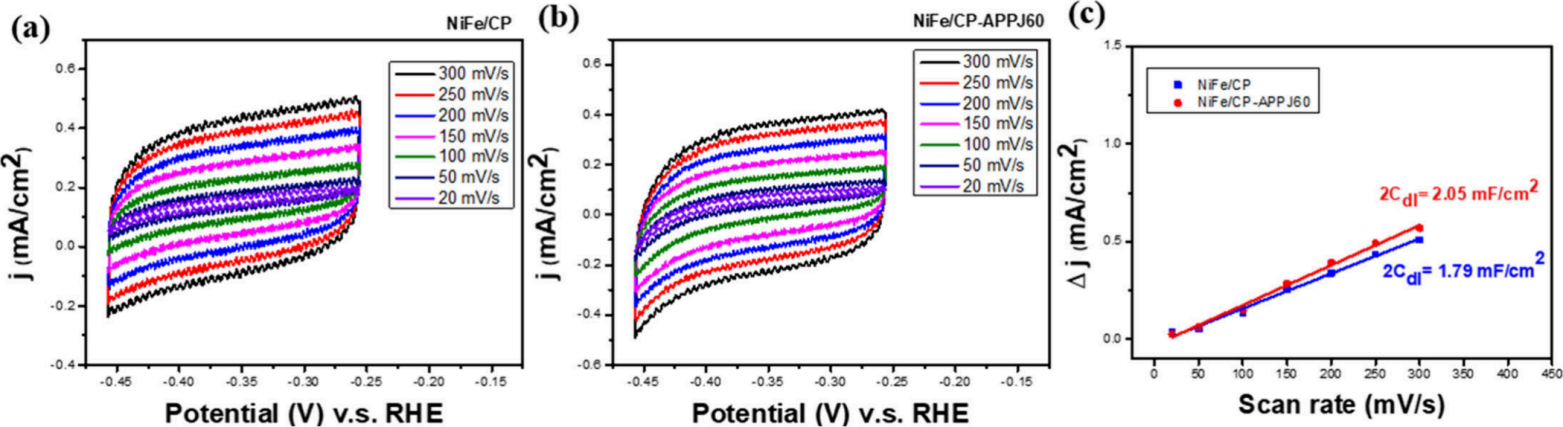
CV measurements of (a) NiFe/CP and (b) NiFe/CP-APPJ60 under different scan rates and (c) calculation of electric double-layer capacitance.

The CV measurement was used to calculate the double-layer capacitance (2*C*_dl_) values to infer the electrochemical active surface area (ECSA).^62−64^ As shown in Figure 12 and Table 4, the 2*C*_dl_ value of NiFe/CP-APPJ60 is 2.05 mF/cm^2^ and that of NiFe/CP is 1.79 mF/cm^2^. The larger 2*C*_dl_ value of NiFe/CP-APPJ60 indicates that it has an ECSA larger than that of NiFe/CP.

**Figure 13:**
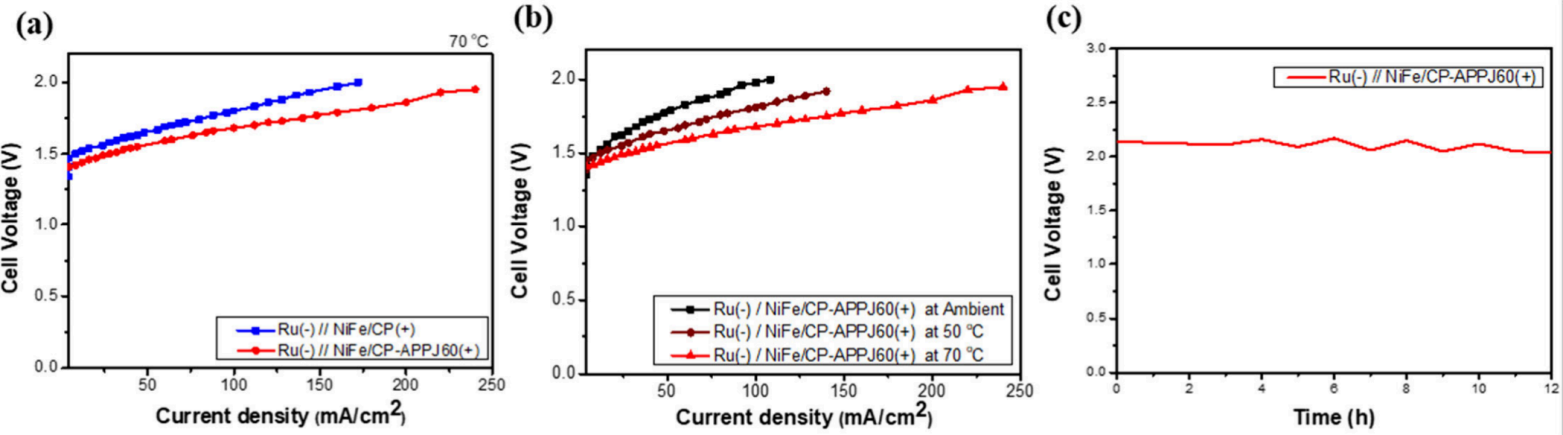
(a) Cell voltage and current density curves in the electrolyzer, with Ru(−)//NiFe/CP(+) and Ru(−)//NiFe/CP-APPJ60(+) compared, (b) cell voltage–current density of electrolyzers at different temperatures of Ru(−)//NiFe/CP-APPJ60(+), and (c) stability test of Ru(−)//NiFe/CP-APPJ60(+).

The custom-made AEMWE was used to test the GDLs of NiFe/CP and NiFe/CP-APPJ60. The AEMWE was used to measure the relationship between the voltage and current density to realize the practical large-scale usage of hydrogen production. In the AEMWE, Ru was deposited on CP as the cathode GDL and NiFe/CP and NiFe/CP-APPJ60 were used in the anode GDL. The two combinations tested in the AEMWE were denoted as Ru(−)//NiFe/CP and Ru(−)//NiFe/CP-APPJ60. The performance of the electrolyzer was quantified by the parameters of energy efficiency (η) and specific energy consumption. The energy efficiency (η) is computed as ,^65^ where *E*_H_2__ is the chemical energy of the produced hydrogen gas, *Q* is the electrical energy input during hydrogen production, *P*_H_2__ is the amount of hydrogen gas produced, 11.7 J is the energy per milliliter of hydrogen gas, *I* is the electrode current, and *V*_ps_ is the power supply voltage. Additionally, the specific energy consumption for generating both a cubic meter and a kilogram of hydrogen gas was calculated. In Tables 5 and 6, the cell voltage refers to the voltage used by the AEMWE for water electrolysis reactions, while the power supply voltage includes both the voltage of the external circuit and the voltage applied in the AEMWE. The value of cell voltage is used to analyze the relationship between voltage and current during water electrolysis reactions. On the other hand, the data of power supply voltage are used to calculate the hydrogen production efficiency and specific energy consumption of the overall electrolysis system. Figure 13a shows a comparison of the performance of Ru(−)//NiFe/CP and Ru(−)//NiFe/CP-APPJ60 at an operating temperature of 70 °C. From the data in Table 5, with NiFe/CP-APPJ60 as the anode, the cell voltage was 1.7 V at a current density of 100 mA/cm^2^, whereas that in the case without APPJ treatment was 1.8 V. Ru(−)//NiFe/CP-APPJ60 presented a higher energy efficiency (85.66%) than that of Ru(−)//NiFe/CP (79.68%) and a lower specific energy consumption for producing per unit of hydrogen gas. The results indicate that Ru(−)//NiFe/CP-APPJ60 needs a lower voltage supply and, thus, has lower energy consumption than Ru(−)//NiFe/CP-APPJ60 at the same current density.

**Table 5:** Performance of the Electrolyzer at 70 °C

current density at 100 mA/cm^2^	cell voltage (V)	power supply voltage (V)	H_2_ production rate (experimental) (mL/min)	energy efficiency, η (%)	specific energy consumption (volume) (kWh/m^3^)	specific energy consumption (weight) (kWh/kg)
Ru(−)//NiFe/CP(+)	1.8	1.86	19	79.68	4.08	45.68
Ru(−)//NiFe/CP-APPJ60(+)	1.7	1.73	19	85.66	3.79	42.49

**Table 6:** Performance of Ru(−)//NiFe/CP-APPJ60(+) in the Electrolyzer at Different Temperatures

current density at 100 mA/cm^2^ (°C)	cell voltage (V)	power supply voltage (V)	H_2_ production rate (experimental) (mL/min)	energy efficiency, η (%)	specific energy consumption (volume) (kWh/m^3^)	specific energy consumption (weight) (kWh/kg)
room temperature	1.98	2.03	19	73	4.45	49.86
50	1.81	1.86	19	79.68	4.08	45.68
70	1.7	1.73	19	85.66	3.79	42.49

According to previous research, the higher operating temperature may accelerate the reaction rate of water electrolysis. Further, the voltage that must be supplied to the cell decreases at higher temperatures owing to the lower equivalent electrical impedance of the cell.^66,67^ The conductivity of alkaline electrolytes also increases with the higher temperature.^68,69^ Consequently, the performance of Ru(−)//NiFe/CP-APPJ60(+) at different operating temperatures was tested in this study, as shown in Figure 13b. On the basis of the results of Table 6, as the temperature increases, the cell voltage for a current density of 100 mA/cm^2^ decreases, indicating better energy efficiency and lower specific energy consumption. Moreover, the stability of Ru(−)//NiFe/CP-APPJ60(+) was measured for 12 h under a current density of 100 mA/cm^2^, as shown in Figure 13c. The cell voltage decreased from 2.14 to 2.04 V, representing a change of approximately −4.6% over the continuous 12 h testing period.

## Conclusion

This study demonstrated the effect of APPJ treatment on ED NiFe/CP and the potential application of the AEMWE. APPJ treatment afforded a higher surface energy and created more oxygen-containing species, and it further enhanced the hydrophilicity. Ni and Fe presented the chemical bonds of carbides in as-deposited ED NiFe/CP; after 60 s of APPJ treatment, the amount of carbide bonds was reduced, and more oxygen-containing species were introduced in NiFe/CP-APPJ60. The change of carbide bonding and the high-energy particles in the plasma also caused higher levels of disorder and more defects in the CP substrate. Between Ni and Fe, Ni showed a more significant change in the oxidation state after APPJ treatment. Owing to the presence of more oxide bonds, APPJ-treated NiFe/CP-APPJ60 presented better OER catalyst performance at a higher current density. A custom-made AEMWE instrument was also tested. Ru(−)//NiFe/CP-APPJ60(+) showed a higher energy efficiency (85.66% at 70 °C) and lower specific energy consumption than those in the case without APPJ treatment. In terms of stability measurements, both electrochemical tests and AEMWE measurements indicate that APPJ post-treatment does not significantly damage the material or gas diffusion layer nor does it cause material instability.

## Supporting Information Available

XRD and EDS analyses, electrochemical characteristics of the cathode material (Ru on CP) used in AEMWE, and comparative data of NiFe/CP and NiFe/CP-APPJ60 used in AEMWE at different operation temperatures (PDF)
