# Supplementary material for: Direct Current Pulse Atmospheric Pressure Plasma Jet Treatment on Electrochemically Deposited NiFe/Carbon Paper and Its Potential Application in an Anion-Exchange Membrane Water Electrolyzer
Source: Langmuir. 2024 Jul 1;40(29):14978–89. doi: 10.1021/acs.langmuir.4c01169 (PMC11271009; doi:10.1021/acs.langmuir.4c01169)
Supplement: Supplementary file 1 — la4c01169_si_001.pdf [file la4c01169_si_001.pdf]

Supporting Information

DC-pulse atmospheric-pressure plasma jet (APPJ) treatment on electrochemically deposited NiFe/carbon paper (CP) and its potential application in anion exchange membrane water electrolyzer (AEMWE)

Shuo-En Yu[1], Yu-Lun Su[2], I-Chih Ni[3] , Yi-Cheng Chuang[4], Cheng-Che Hsu[5], Chih-I Wu[1,3], Yong-Song Chen[4], I-Chun Cheng[3], Jian-Zhang Chen[1,2,6,*]

[1] Graduate School of Advanced Technology, National Taiwan University, Taipei City 106319, Taiwan

[2] Institute of Applied Mechanics, National Taiwan University, Taipei City 106319, Taiwan

[3] Graduate Institute of Photonics and Optoelectronics and Department of Electrical Engineering, National Taiwan University, Taipei City 106319, Taiwan

[4] Department of Mechanical Engineering and Advanced Institute of Manufacturing with High-Tech Innovations, National Chung Cheng University, Chiayi County 621301, Taiwan

[5] Department of Chemical Engineering, National Taiwan University, Taipei City 10617, Taiwan

[6] Advanced Research Center for Green Materials Science and Technology, National Taiwan University, Taipei City 106319, Taiwan

* Correspondence: jchen@ntu.edu.tw (J.Z.C.); Tel.: +886-2-33665694 (J.Z.C.)

List

1. **Table S1. Mass loading of each sample**
2. **Figure S1. Single point EDS of (a) NiFe/CP and (b) NiFe/CP-APPJ60**
3. **Figure S2. XRD result of each sample**
4. **Figure S3. Curve of current density-cell voltage of Ru(-)//NiFe/CP and Ru(-)//NiFe/CP-APPJ60 at (a) room temperature, (b) 50 °C, and (c) 70 °C**
5. **Table S2. Calculated parameters of Ru(-) // NiFe/CP (without APPJ treatment) under different operating temperatures**
6. **Figure S3. Electrochemical measurement of Ru (a) LSV HER polarization curves in 1 M KOH. (b) Tafel slope plots. (c) Nyquist plots at an overpotential of 400 mV versus RHE.**
7. **Table S3. Overpotential at different current densities (unit: mV)**
8. **Table S4. EIS analysis of CP and Ru**

Table S1. Mass loading of each sample

Sample	**Mass loading**
**CP**	-
**NiFe/CP**	~1.8 mg/cm^2
**NiFe/CP-APPJ60**	~1.4 mg/ cm^2
**Ru**	~1 mg/ cm^2

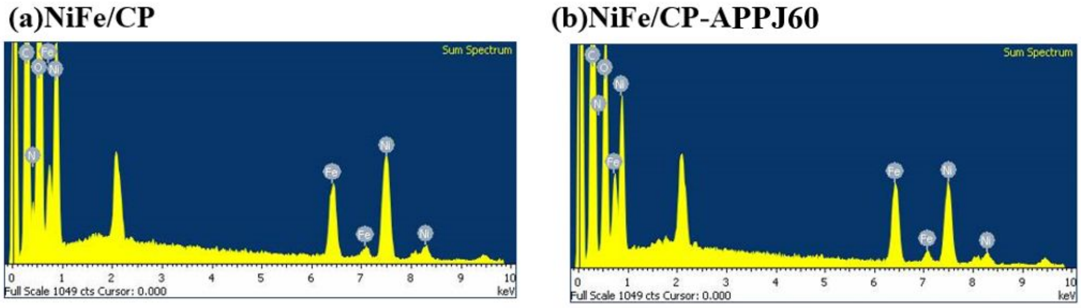

Figure S1. Single point EDS of (a) NiFe/CP and (b) NiFe/CP-APPJ60

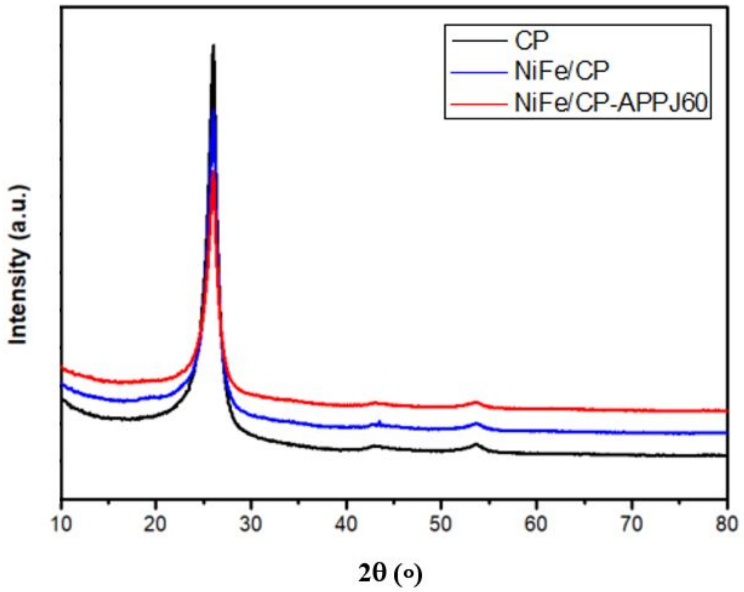

Figure S2. XRD result of each sample

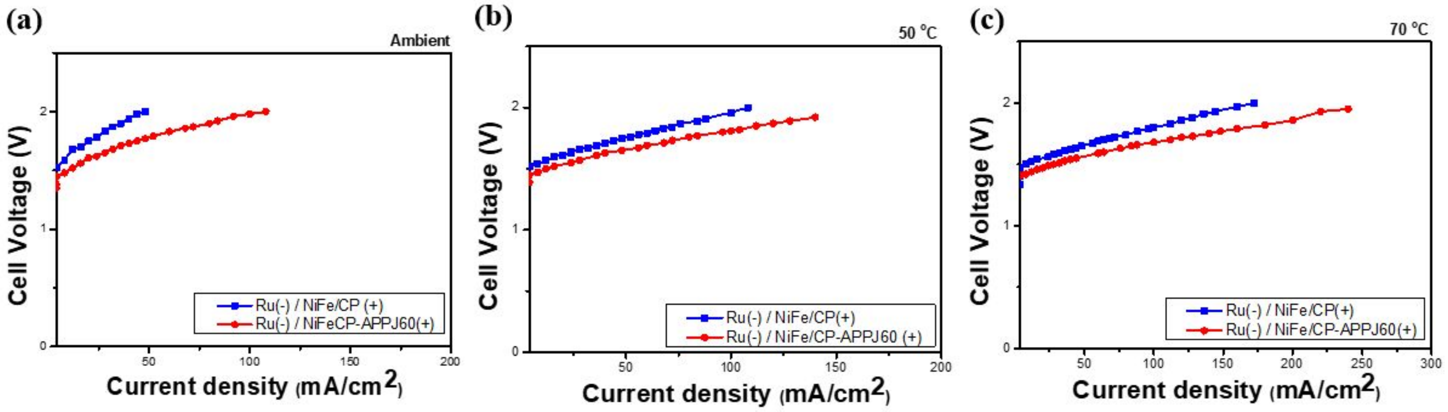

Figure S3. Curve of current density-cell voltage of Ru(-)//NiFe/CP and Ru(-)//NiFe/CP-APPJ60 at (a) room temperature, (b) 50 °C, and (c) 70 °C.

Table S2. Calculated parameters of Ru(-) // NiFe/CP (without APPJ treatment) under different operating temperatures

Current density @ 100 mA/cm²	Cell voltage	Power supply voltage	H_2 production rate (experimental)	Energy efficiency (η)	Specific energy consumption (volume)	Specific energy consumption (weight)
**℃**	**V**	**V**	**ml/min**	**%**	**kWh/m³**	**kWh/kg**
**50 ℃**	1.96	2.01	19	73.73	4.41	49.37
**70 ℃**	1.8	1.86	19	79.68	4.08	45.68

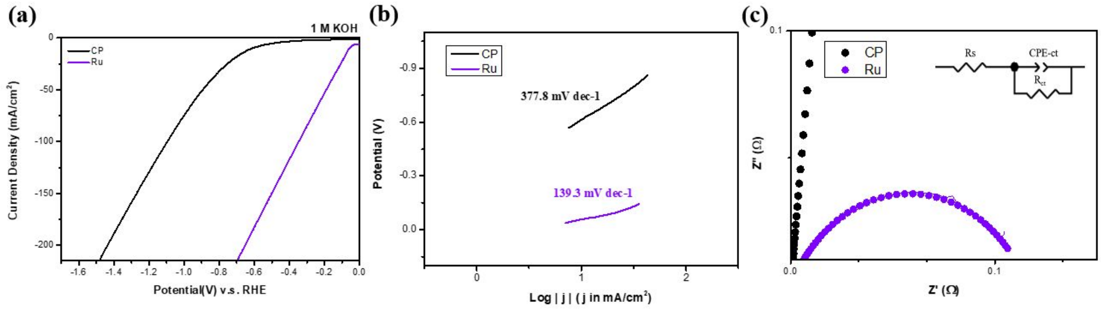

Figure S3. Electrochemical measurement of Ru (a) LSV HER polarization curves in 1 M KOH. (b) Tafel slope plots. (c) Nyquist plots at an overpotential of 400 mV versus RHE.

Table S3. Overpotential at different current densities (unit: mV)

**Electrocatalyst**	**@10mA/cm^2**	**@50mA/cm^2**	**@100mA/cm^2**	**@150mA/cm^2**	**@200mA/cm^2**
**CP**	613.9	-	-	-	-
**Ru**	60.0	192.2	348.7	499.8	651.8

Table S4. EIS analysis of CP and Ru

Sample	**R_{ct}**
**CP**	85.56 Ω
**Ru**	0.86 Ω
